# The relationship between university students’ goal orientation and academic achievement. The mediating role of motivational components and the moderating role of achievement emotions

**DOI:** 10.3389/fpsyg.2023.1296346

**Published:** 2024-01-23

**Authors:** Florin-Vasile Frumos, Roxana Leonte, Octav Sorin Candel, Laura Ciochină-Carasevici, Roxana Ghiaţău, Camelia Onu

**Affiliations:** Faculty of Psychology and Education Sciences, Alexandru Ioan Cuza University, Iaşi, Romania

**Keywords:** achievement emotions, goal orientation, motivational components, academic achievement, moderated mediation analysis, university students

## Abstract

The present study aims to expand the understanding of the role played by achievement emotions in the learning process and academic achievement of university students. We investigated how achievement emotions moderate the direct and indirect associations between mastery and performance goal orientation and academic achievement. Also, we used as mediators the motivational components from Pintrich and De Groot’s theoretical framework of motivation and learning strategies. 274 Romanian university students (M_*age*_ = 20.23, 84.7% women) participated in the study. Moderated mediation analyses indicated that self-efficacy was the only significant mediator, and this relationship was moderated by hope, pride and hopelessness. In addition, the links between mastery and performance approach goals and motivational components are stronger when the positive emotions are higher and the negative ones are lower. Mastery avoidance goals were linked with high scores of motivational components at higher levels of negative and lower levels of positive achievement emotions, whereas the association of avoidance goals with motivational components was moderated by two positive emotions (pride and enjoyment). The patterns derived from the moderating role of achievement emotions in the relationships between goal orientation, motivational components and academic achievement, alongside several inconsistent results and implications in theory and education, are discussed.

## 1 Introduction

Students’ academic achievement depends on a diversity of interacting psychological variables. Among these, some of the most important are learning goals orientation (Elliot and McGregor, 2001; Keys et al., 2012; Dinger and Dickhäuser, 2013; Cerasoli and Ford, 2014), motivational components (Pintrich and De Groot, 1990; Stegers-Jager et al., 2012; Muwonge et al., 2019; Bai and Wang, 2023), and the emotions experienced while studying (Pekrun, 2011). Considering goal orientation theory of achievement motivation (Elliot and McGregor, 2001), mastery approach and performance approach goal orientations positively influence the academic results of students (Eum and Rice, 2011; Darnon et al., 2018), whereas mastery avoidance and performance avoidance goal orientation negatively predict this academic output (Elliot and Church, 1997; Baranik et al., 2010). However, learning goal orientations does not always directly predict academic achievement, their effect being mediated by others motivational factors (Honicke et al., 2019); components pertaining from the students’ learning motivation such as academic self-efficacy and effort regulation show medium-size correlations with academic achievement (Richardson et al., 2012). Therefore, components as expectancies for success and subjective value of the learning tasks (Eccles, 1983) interact with goal orientation and influence academic results. In this regard, the model of self-regulated learning of Pintrich (2000a) indicate that goal orientation and self-efficacy represent essential motivational variables that influence academic achievement.

The importance of emotions in human life is widely recognized and investigated from psychological, but also from a broader philosophical perspective (de Sousa, 1979). Emotions are important because they made salient for us various dimensions of things (Elgin, 2008), for instance, emotions facilitate evaluative understanding (Brady, 2013), relate with the theories and beliefs they hold about knowing (Hofer and Pintrich, 1997) and represent epistemic forces toward the truth (Candiotto, 2020). Also, emotional cognition is useful for understanding thinking in law, religion and science (Thagard, 2006).

As complex fenomena, emotions involve affective, cognitive, physiological, motivational and behavioral components (Scherer, 2009). We encounter a large spectrum of emotions associated with learning: moods, that represent diffuse affective states as feeling joyful, angry or fearful (Pekrun, 2011); feeling of certainty or doubt (de Sousa, 2009) and other epistemic emotions, related to knowledge and knowing (curiosity or confusion; Pekrun, 2011), achievement emotions, linked with learning activities, as enjoyment of learning, but also boredom related with learning tasks; and content-related or topic emotions, as worrying about a protagonist wen reading a novel; social emotions, as admiration, envy or shame (Pekrun, 2011).

Research on emotions in academic settings significantly evolved in the last decades, and literature on achievement emotions clearly indicate they are related with academic achievement and motivational variables. The control-value theory of achievement emotions, (Pekrun et al., 2006) claim that students’ emotions while study impacts their self-regulated learning, motivation and academic achievement. Further, achievement emotions affect psychological well-being, happiness, and life satisfaction (Pekrun, 2006), problem-solving ability (Lee and Chei, 2020), learning persistence (Tang et al., 2021), and can provide the motivational and physiological energy for engaging in future actions (Pekrun et al., 2002). Hence, the interaction between learning goal orientation and expectancies or value motivational variables happen into a broader learning context, where specific discrete emotion as enjoyment, boredom or hope modulate the intensity and the nature of relationships and their impact on academic achievement.

However, despite a great number of studies exploring the single and combined effect of learning goals orientation, motivational components as expectation for success and subjective task value, and achievement emotion on academic outputs, the specific mechanisms of interactions between these variables are far from being clearly understood. It is a gap in understanding the specific roles that achievement emotions play in interactions between goal orientation and others motivational variables, further influencing academic achievement; as achievement emotions represents background elements of the broader learning context, it is reasonable to consider these emotions modulate relationships between goal orientations and motivational components, rather than directly influence academic achievement. Also, the specific moderating effect of different achievement emotions in these relationships worth to be known.

In sum, this study proposes to further shed light on the relationships between learning goal orientation, motivational components and achievement emotions and how they related with academic achievement in university students. More specifically, our study first aims to evaluate the mediating role of motivational components between goal orientation and academic achievement. Second, we wanted to explore the ways in which achievement emotions felt when studying moderate the direct and indirect associations between specific goals orientations and academic achievement through motivational components. This approach may contribute to a better understanding of the underlying mechanisms and dynamics of motivational and affective factors contributing to the academic achievement of university students.

The present study adds to the existing literature with a comprehensive analysis of the role played by achievement emotions (Pekrun, 2011) as moderators of the relationships between goal orientation (Elliot and McGregor, 2001), expectancy, value and affective motivational components (Pintrich et al., 1991), and academic achievement. Pekrun et al. (2002) emphasize that the results of the studies on the motivational components, goal orientation and achievement emotions should be more useful to counseling and educational intervention aiming to improve students’ learning process. Clarifying this role may further substantiate interventions for improving learning and teaching in university settings (Pekrun et al., 2006; Daniels et al., 2009; Fritea and Fritea, 2013).

### 1.1 Goal orientation and academic achievement

Achievement motivation literature developed from two meanings of competence: as absolute, intrapersonal (mastery), and normative, interpersonal (performance), further involving two types of achievement goals (Elliot, 1997): mastery goal orientation and performance goal orientation respectively (Dweck, 1986). A mastery goal-oriented subject is motivated to develop his or her own competence, through mastering the learning task, whereas a performance goal-oriented learner is focused on demonstrating competence to others (Elliot and McGregor, 2001).

Elliot and Church (1997) proposed that mastery-performance dichotomy of achievement goals should be revised to include the distinction between approach and avoidance motivation, by addition of valence dimension to the performance goal orientation. The trichotomous goal framework keep unchanged the mastery goal orientation, but split the performance goal orientation in two subcategories: performance approach and performance avoidance goal orientation, according with valence (positive or negative) dimension. The performance approach goal orientation reflects the positive, desirable possibility of success, whereas performance avoidance goal orientation reflects the undesirable possibility of failure.

Further extension of the trichotomous framework of achievement goal orientation (Elliot and McGregor, 2001) additionally bifurcated mastery approach goal orientation taking into account the same valence dimension. This conceptual framework is known as the 2 × 2 model of goal orientation: mastery-approach, mastery-avoidance, performance-approach and performance-avoidance goals. A mastery avoidance goal-oriented subjects’ focus is on striving on avoid misunderstanding, not losing skills or nor performing worse than before (Elliot and McGregor, 2001). In our study, we used this 2 × 2 model, which received support as being the most effective in explaining learning outcomes (Huang, 2012).

The relationship between goal orientation and academic achievement was tested in various studies. Directing the goals toward a good mastery of the content (mastery-approach goal) positively influences the academic achievement of students (Darnon et al., 2018; Suprayogi et al., 2019; Alhadabi and Karpinski, 2020), due to their focus on the development of knowledge, competences, skills and abilities (Diaconu-Gherasim and Mãirean, 2016). At the same time, performance-approach goals, manifested by demonstrating competences and overcoming others in order to receive appreciation for their results (Pintrich, 2000b; Diaconu-Gherasim and Mãirean, 2016), also have positive effects on academic achievement (Goraya and Hasan, 2012; Darnon et al., 2018; Suprayogi et al., 2019). However, mastery avoidance and performance avoidance goal orientations negatively influence the academic achievement of students (Baranik et al., 2010; Luo et al., 2013). The lack of confidence in one’s own abilities and the concern to avoid situations that could prevent the full understanding of the content is reflected by the mastery-avoidance goal orientation (Elliot and Church, 1997; Baranik et al., 2010; Hulleman et al., 2010; Alhadabi and Karpinski, 2020), whereas the prevention of negative judgments by avoiding tasks that could reveal the lack of skills or competences (Pintrich, 2000b; McCollum, 2004) is reflected by performance-avoidance goal orientation. In order to achieve academic success, students can adopt and pursue a combination of learning goal orientations (Cho et al., 2011; Dull et al., 2015).

Nevertheless, the results of previous studies suggest that the link between goal orientations and academic achievement is not straightforward, some studies indicating that these relationships could be mediated by different factors (Bipp and van Dam, 2014; King and McInerney, 2014; Zhou and Wang, 2019). For instance, the relationship between mastery goals and academic achievement may be mediated by deep-processing strategies (Greene and Miller’s 1996) or effort expenditure (Dupeyrat and Mariné, 2005). For this study, we tested motivational components (Pintrich et al., 1991) as mediators between goal orientation and academic achievement.

### 1.2 The mediating role of motivational components

Regulation of learning involves, on the one hand, managing one’s own motivational beliefs, such as self-efficacy and task-value belief (Wolters, 1998, 2003; Pintrich, 2000a), and on the other hand, controlling one’s learning strategies, thoughts and actions that influence choices, effort and persistence in academic tasks, in order to achieve good academic results (Wolters, 2003; Zimmerman and Schunk, 2011). Among learning motivation theories, one of the most influential is expectancy-value theory of Eccles (1983), Eccles and Wigfield (2002). This theory states students’ choice and engagement in learning task is determined by two subjective, task-specific motivational orientations and beliefs (Gaspard et al., 2018): the expectancy that they can succeed in that task (“Can I do it?”) and (b) the value of task (“Do I want to do it?”). The expectancy dimension about success depicts individual’ s beliefs about how well will accomplish tasks and is conceptually related with academic self-concept (Marsh, 2006). The learners’ orientations and beliefs about value dimension involve four subjective task value dimensions: attainment value or the personal importance to doing well a task; intrinsic value as interest or enjoyment of subject doing the task; utility value related with current or future subject’s learning goals, and cost value, representing negative aspects as anxiety of failure, effort required or lost opportunities when one choose a specific learning task (Eccles and Wigfield, 2002; Tang et al., 2022). Recent theoretical synthesis reveals the Situated Expectancy-Value Theory (SEVT, Wigfield and Eccles, 2020), which represents the original expectancy-value theory completed with the socio-cultural dimension, has been utilized more than any of the other theories in motivation studies with longitudinal design (Anderman, 2020).

As Pintrich and De Groot (1990) states, the theoretical framework that conceptualize students’ motivation is the general, original expectancy-value model of motivation (Eccles, 1983). The six motivational components detailed by Pintrich and De Groot (1990) encompass three subcategories, each with specific dimensions as follow: (1) three value components: intrinsic goal orientation (engagement in a task constitutes itself a goal and appears due to interest, curiosity and desire for knowledge); extrinsic goal orientation (the motivation for engaging in academic tasks is external in nature, based on, among others, grades, rewards, positive evaluation or competition); task value (assumes the importance, usefulness and interest given to the learning material); (2) two expectancy components, representing motivational beliefs: control of learning beliefs (refers to the belief that good results are consequences of one’s own effort in learning), and self-efficacy (involves self-assessment of one’s own capabilities and confidence in one’s own skills); (3) one affective component, namely test anxiety, with its cognitive (negative thoughts or concerns that could affect performance), and affective (increased anxiety and worry) aspects (Pintrich et al., 1991).

Motivational components presented above were linked to both academic achievement and goal orientation. Previous studies identified positive correlations between self-efficacy, intrinsic goals orientation and academic achievement (Kosnin, 2007; Kitsantas et al., 2008; Al Khatib, 2010; Trautner and Schwinger, 2020). The link between goal orientation and different motivational components also received strong support. Mastery-approach goals were related to intrinsic motivation, due to the students’ positive attitude and higher level of engagement in academic tasks (Elliot, 2005; Kaplan and Maehr, 2007), while performance-avoidance goals were associated to lower intrinsic motivational orientation (Shi, 2021). Other studies indicate that mastery and performance-approach goals are positively related to the task value (Church et al., 2001; Harackiewicz et al., 2002) and both mastery and performance-avoidance goals were positively related to test anxiety (Palos et al., 2019). In a meta-analysis, Payne et al. (2007) found that mastery-approach goals were associated with high self-efficacy, compared to performance-avoidance goals and that mastery-approach goals were related to lower test anxiety compared to performance-approach and avoidance goals. Shi (2021) also found that self-efficacy was positively correlated with mastery-approach goal orientation and negatively with performance-avoidance goals, but no significant relationship was found between self-efficacy and performance-approach goal orientation.

Since motivational components are associated with both learning goals and academic achievement, they may also function as mediators of the relationship between them. In the study by Honicke et al. (2019), academic self-efficacy mediated the relationships between both mastery and performance-approach goal orientation and academic achievement. Magni et al. (2021) found stronger evidence for the mediating role of self-efficacy in the relationship between an approach goal orientation and students’ performance, compared to the one between an avoidance goal orientation and performance. Bandalos et al. (2003) argue that both mastery and performance goal orientations were associated indirectly with achievement through two motivational components: self-efficacy and test anxiety. Other studies also support the mediating role of self-efficacy in the relationship between mastery goal orientation and academic achievement (Coutinho and Neuman, 2008; Olaogun et al., 2022).

However, the studies specifically investigating the mediation effect of Pintrich and De Groot’s (1990) motivational components on the relationship between learning goals and academic achievement are still scarce (Honicke et al., 2019). As previously discussed, self-efficacy and test anxiety received significantly more attention. Thus, several gaps remain in the understanding how the other motivational components mediate the relationship between goal orientation and academic achievement.

### 1.3 The moderating role of achievement emotions

Emotions in academic settings have an important influence on students’ academic achievement, motivation and learning process (Pekrun et al., 2009; Muis et al., 2015). Achievement emotions are defined as emotions experienced by students in learning settings, their intensity may vary according to gender, age, and culture (Camacho-Morles et al., 2021). These emotions were grouped based on different attributes. The first and most evident attribute of achievement emotions is their valence: positive vs. negative, pleasant vs. unpleasant. Enjoyment, pride and hope are felt as pleasant emotions, whereas anger, anxiety, hopelessness, shame and boredom are unpleasant, negative emotions (Pekrun et al., 2002). Secondly, achievement emotions can be classified as being activity-related, focused on the processes of learning in school-related settings, or output-related, focused on the result of these learning activities (Pekrun, 2006; Pekrun et al., 2009). For example, the anger felt when struggling with a difficult task is an activity-related emotion, whereas the hope for success is an output-related emotion. Thirdly, output-related emotions differ based on their temporal dimension: hope for success is an output-related, prospective emotion, whereas pride experienced after an academic success is an output-related, retrospective emotion (Pekrun, 2006). Fourthly, both the activity-related and outcome-related emotions can be further grouped as activating emotions (enjoyment, hope, pride, anxiety, shame and anger) or deactivating emotions (hopelessness and boredom; Pekrun, 2011). These taxonomies can be further combined (e.g., an emotion’s valence combines with its activating-deactivating dimension) resulting in positive activating emotions (hope, enjoyment and pride), positive deactivating emotion (relief), negative activating emotions (anger, anxiety, shame) and negative deactivating emotions (boredom and hopelessness; Pekrun, 2006, 2011).

The control-value theory of achievement emotions (Pekrun et al., 2006) claims that students’ emotions affect the cognitive, motivational, and regulatory processes influencing learning and achievement. However, the link between achievement emotions and academic achievement is not always intuitive. It should be noted that positive (e.g., pleasant) achievement emotions do not always have positive effects on learning outputs, and the negative links to academic achievement do not always appear in the presence of unpleasant achievement emotions. Thus, pleasant emotions are not by default adaptive, and symmetrically, unpleasant emotions are not always maladaptive for learning purposes (Pekrun, 2011). Although the positive valence of achievement emotions usually counts for positive effects on learning activities and outputs, the interaction of others characteristics such as the activating-deactivating dimension or the appraisal of subjective control and subjective value of learning activities may be more relevant for the link between achievement emotions and learning (Pekrun, 2006).

It is also likely that achievement emotions facilitate the use of different learning strategies and promote different styles of regulation of learning, such as the students’ intrinsic motivation to learn (Mega et al., 2014). Moreover, hope and enjoyment are positively, whereas anger, anxiety and boredom are negatively related with study interest, self-regulation and effort as components of self-regulated learning motivational strategies (Pekrun et al., 2002). Considering the valence and activation dimensions of emotions, positive activating emotions such as enjoyment can promote intrinsic and extrinsic motivation and self-regulation, while positively affecting academic achievement. On the contrary, negative deactivating emotions, such as hopelessness and boredom, can reduce motivation and have negative effects on academic results (Pekrun et al., 2011; Tze et al., 2016).

Achievement emotions are related with students’ performance through motivational mechanisms. Students’ achievement emotions influence their self-regulated learning and their motivation, and these, in turn, affect academic achievement (Mega et al., 2014). Positive activating emotions can positively influence performance, by increasing motivation and stimulating flexible learning, while negative deactivating emotions can affect performance by diminishing motivation, distracting attention and superficial solving of tasks (Pekrun et al., 2002; Pekrun et al., 2009; Muis et al., 2015). A recent systematic review suggest positive achievement emotions in online learning contexts may be much more effective than negative ones in improving learners’ motivation, performance and achievement, but in the same time negative activating emotions, such as anxiety and frustration, also positively influence performance of subjects (Wu and Yu, 2022).

Previous results show that there is sufficient evidence for the role of achievement emotion in shaping motivational components and academic achievement. Moreover, as variables involved in broader learning contexts, emotions felt when studying may modulate the associations of different types of learning goals with specific motivational components. Previous studies, although using different outcomes, show that achievement emotions can interact with learning goals, significantly moderating their relationship with teachers’ identity construct (Çetin and Eren, 2022). In this study, we want to go further exploring the moderating role of achievement emotions in the relationships between goal orientation, motivational components and academic achievement.

### 1.4 The present study

Previous studies showed that the academic achievement is determined by learning goal orientation (Darnon et al., 2018), motivational components (Kosnin, 2007; Kitsantas et al., 2008; Trautner and Schwinger, 2020) and achievement emotions (Pekrun et al., 2009; Muis et al., 2015). Several researchers have examined the combined role of these factors on academic achievement, such as learning goal orientation and motivational components (Church et al., 2001; Palos et al., 2019) and learning goal orientation and achievement emotions (Pekrun et al., 2006, 2009).

However, there are still important gaps in the literature. The studies that analyzed the mediating role of motivational variables in the relationship between learning goal orientation and academic achievement focused on academic self-efficacy in particular (Coutinho and Neuman, 2008; Magni et al., 2021). However, the other motivational components received little or no interest at all. In addition, to our knowledge, no study has verified the moderating role of achievement emotions on the relationships between goal orientation, motivation of learning and academic achievement.

Thus, the main objective of our study is to explore whether achievement emotions while study moderate the direct and indirect associations between specific goals orientations and academic achievement through motivational components in learning.

To conclude, we hypothesized the followings:

1. There is a positive association between both mastery-approach and performance-approach goal orientation and academic achievement.
2. There is a negative association between both mastery-avoidance and performance-avoidance goal orientation and academic achievement.
3. Motivational components mediate the relationship between goal orientation and academic achievement.
4. Achievement emotions moderate the relationship between goal orientation, motivational components and academic achievement.
   Specifically, we expected that:
  4.1. Positive emotions increase the positive associations between goal orientation, motivational components and academic achievement.
  4.2. Positive emotions decrease the negative associations between goal orientation, motivational components and academic achievement.
  4.3. Negative emotions decrease the positive associations between goal orientation, motivational components and academic achievement.
  4.4. Negative emotions increase the negative associations between goal orientation, motivational components and academic achievement.

## 2 Materials and methods

### 2.1 Participants and procedure

The participants in the study were 372 students enrolled in two bachelor programs at a large north-eastern Romanian Institution. However, due to missing data, only 274 participants were retained for the current study. From these, 139 (50.7%) were enrolled in a Psychology program and 135 (49.3%) were enrolled in a Social Sciences program. The students had a mean age of 20.23 years, with a SD of 3.62. 42 participants (15.3%) identified themselves as men and 232 (84.7%) as women. 141 participants (51.5%) lived in urban areas, while 133 participants (48.5%) lived in rural areas. All the students were recruited in a Pedagogy course. Their involvement in the study was voluntary and rewarded with course credit. The participation was anonymous.

The study was approved by The Ethics Committee of the Faculty of Psychology and Education Sciences, at the “Alexandru Ioan Cuza” University of Iasi. The participants who agreed to the take part in the study completed the questionnaires in a pen-and-paper format, in the classroom.

### 2.2 Measures

#### 2.2.1 Goal orientation

The Achievement Goal Questionnaire (AGQ; Elliot and McGregor, 2001) was used to measure the four types of learning goal orientation: mastery-approach goals (3 items; e.g., “My goal is to learn as much as possible”), mastery-avoidance goals (3 items; e.g., “My aim is to avoid learning less than I possibly could”), performance-approach goals (3 items; e.g., “My aim is to perform well relative to other students”) and performance-avoidance goals (3 items; e.g., “My aim is to avoid doing worse than other students”). Participants responded to the items on a seven-point scale (1 = not at all true of me – 7 = very true of me). Reliability coefficients for each scale were good and are included in Table 1.

**TABLE 1:** Means, standard deviation, minimum, maximum and Cronbach’s alpha for the variables included in the study.

	M	SD	Min	Max	Cronbach’s alpha
Academic achievement	8.64	0.73	5.00	10.00	–
MAP	16.55	3.77	3.00	21.00	0.86
MAV	14.92	4.02	3.00	21.00	0.72
PAP	14.05	5.23	3.00	21.00	0.94
PAV	13.93	5.28	3.00	21.00	0.92
IGO	19.90	5.24	4.00	28.00	0.78
EGO	18.76	6.32	4.00	28.00	0.86
Task value	32.85	6.65	6.00	42.00	0.89
CLB	23.43	3.89	10.00	28.00	0.75
Self-efficacy	38.99	9.54	8.00	56.00	0.92
Test anxiety	20.11	7.86	5.00	35.00	0.85
Enjoyment during course studying	15.47	3.18	4.00	20.00	0.85
Hope during course studying	15.47	3.23	4.00	20.00	0.88
Pride during course studying	16.49	3.15	5.00	20.00	0.85
Anger during course studying	7.33	3.23	4.00	20.00	0.85
Anxiety during course studying	10.37	3.77	4.00	20.00	0.76
Shame during course studying	7.58	3.86	4.00	19.00	0.85
Hopelessness during course studying	6.87	3.74	4.00	20.00	0.89
Boredom during course studying	7.91	3.82	4.00	20.00	0.90

MAP, Mastery-Approach goals orientation; MAV, Mastery-Avoidance goals orientation; PAP, Performance-Approach goals orientation; PAV, Performance-Avoidance goals orientation; IGO, Intrinsic Goals Orientation; EGO, Extrinsic Goals Orientation; CLB, Control of Learning Beliefs.

#### 2.2.2 Motivational components

The Motivated Strategies for Learning Questionnaire (MSLQ –Pintrich et al., 1991) has been widely used to investigate students’ motivational components, its validity being shown by numerous studies (Kosnin, 2007; Roth et al., 2016; Tabatabaei et al., 2017). MSLQ was used in the present study to measure the six motivational components: intrinsic goal orientation (4 items; e.g., “The most satisfying thing for me is trying to understand the content as thoroughly as possible”); extrinsic goal orientation (4 items; e.g., “Getting a good grade is the most satisfying thing for me right now”); task value (6 items; e.g., “I am very interested in the content area of the courses”), control of learning belief (4 items; e.g., “If I try hard enough, then I will understand the course material”), self-efficacy (8 items; e.g., “I’m certain I can understand the most difficult material presented at courses”) and test anxiety (5 items; e.g., “When I take a test I think about how poorly I am doing compared with other students”). The items were measured on a 7-point Likert scale (1 = not at all true; 7 = very true). Reliability coefficients, means and standard deviations are included in Table 1.

#### 2.2.3 Achievement emotions

The Achievement Emotions Questionnaire (AEQ, Pekrun et al., 2011) is a well-established instrument for measuring achievement emotions in educational research (Bieleke et al., 2021). The original AEQ scale was large and unsuitable for use in conditions of brief administration time, thus a shorten version AEQ-S was developed and validated, showing satisfactory reliability and good correlation with the original AEQ scale (Bieleke et al., 2021). AEQ-S comprises items for the four components of each emotion considered in the AEQ (i.e., affective, cognitive, motivational, and physiological – see Table 2), in three learning settings (class, learning and test-related settings), resulting 96 items in eight scales.

**TABLE 2:** Item examples for achievement emotions measured with AEQ-S in learning-related settings.

	Components	Items
Pride	Affective	I’m proud of myself.
	Cognitive	I think I can be proud of my accomplishments at studying.
	Motivational	Because I want to be proud of my accomplishments, I am very motivated.
	Physiological	When I excel at my work, I swell with pride.
Anxiety	Affective	I get tense and nervous while studying.
	Cognitive	I worry whether I’m able to cope with all my work.
	Motivational	While studying I feel like distracting myself in order to reduce my anxiety.
	Physiological	Worry about not completing the material makes me sweat.

In our study, we used AEQ-S in learning-related setting, for eight emotions: enjoyment, hope, pride, anger, anxiety, shame, hopelessness, and boredom. Therefore, we used 32 items grouped in eight scales, measured on 5-point Likert scale (1 = strongly disagree to 5 = strongly agree). Reliability coefficients, means and standard deviations are included in Table 1.

#### 2.2.4 Academic achievement

Student’s academic achievement was measured based on their self-reported grade point average attained in the previous academic year.

### 2.3 Statistical analyses

The preliminary and the correlation analyses were conducted using the IBM SPSS 20 statistical software. To test the normality of the distributions we computed the Skewness and Kurtosis measures. Normal distributions were presented for all variables. To test the proposed moderated mediation models, we used Model 8 from Process, an SPSS macro (Hayes, 2013). For the mediation, bootstrapping with 5,000 re-samples was used to obtain parameter estimates of the specific indirect effects. The 95% confidence intervals (CIs) were used to determine whether these effects were statistically significant: if the 95% CI did not contain zero, then the indirect effect was considered statistically significant and mediation was demonstrated. For the moderation, we computed simple slope analyses to test the conditional effects of the predictor at low (16th percentile), medium (50th percentile), and high (84th percentile) levels of the moderator. All the variables included in the interactions were centered before the analyses. Because the Process macro does not compute standardized coefficients for the models that include moderation, unstandardized coefficients were reported for the analyses.

## 3 Results

### 3.1 Preliminary analyses

The means, standard deviations, minimum and maximum and the Cronbach’s Alpha coefficient for all the variables considered in the study are included in Table 1.

### 3.2 Correlation analyses

Given than the data were normally distributed, we used Pearson-product correlations. The analyses showed that academic achievement was significantly and positively associated with all the four types of academic goals (see Table 3). Also, it was significantly and positively associated with extrinsic goal orientation and self-efficacy. However, the effect sizes for the all the significant correlations were small. Academic achievement was also positively and significantly related to feeling enjoyment, hope and pride during course studying, and negatively related to feeling anger, anxiety, shame and hopelessness. Again, the effect sizes were small.

**TABLE 3:** Correlational analysis for the variables included in the study.

	1	2	3	4	5	6	7	8	9	10	11	12	13	14	15	16	17	18
1. Academic achievement	–																	
2. MAP	0.21**	–																
3. MAV	0.22**	0.54**	–															
4. PAP	0.23**	0.44**	0.37**	–														
5. PAV	0.18**	0.41**	0.42**	0.89**	–													
6. IGO	0.01	0.36**	0.18**	0.02	0.02	–												
7. EGO	0.18**	0.41**	0.32**	0.80**	0.76**	0.02	–											
8. Task value	0.09	0.62**	0.35**	0.25**	0.24**	0.40**	0.27**	–										
9. CLB	0.04	0.37**	0.18**	0.11*	0.12*	0.17**	0.12*	0.32**	–									
10. Self-efficacy	0.28**	0.55**	0.36**	0.32**	0.25**	0.28**	0.29**	0.52**	0.23**	–								
11. Test anxiety	0.01	0.10	0.09	0.24**	0.31**	-0.04	0.30**	-0.08	-0.04	-0.23**	–							
12. Enjoyment	0.18**	0.39**	0.25**	0.24**	0.20**	0.26**	0.15*	0.33**	0.06	0.39**	-0.15*	–						
13. Hope	0.19**	0.37**	0.26**	0.29**	0.28**	0.20**	0.20**	0.40**	0.07	0.44**	-0.20**	0.67**	–					
14. Pride	0.18**	0.34**	0.19**	0.29**	0.29**	0.12*	0.28**	0.26**	0.07	0.38**	-0.10	0.55**	0.68**	–				
15. Anger	-0.16**	-0.20**	-0.11	-0.05	-0.03	-0.14*	-0.03	-0.17**	-0.09	-0.22**	0.24**	-0.42**	-0.42**	-0.35**	–			
16. Anxiety	-0.12*	-0.09	-0.02	-0.01	0.03	-0.10	0.02	-0.17**	-0.10	-0.23**	0.46**	-0.31**	-0.40**	-0.39**	0.60**	–		
17. Shame	-0.20**	-0.11	-0.03	-0.03	-0.04	-0.05	0.01	-0.14*	-0.01	-0.23**	0.33**	-0.35**	-0.41**	-0.47**	0.45**	0.56**	–	
18. Hopelessness	-0.22**	-0.19**	-0.14*	-0.13*	-0.11	-0.09	-0.06	-0.19**	-0.07	-0.30**	0.31**	-0.42**	-0.55**	-0.58**	0.57**	0.62**	0.72**	–
19. Boredom	-0.11	-0.23**	-0.13*	-0.11	-0.07	-0.13*	-0.04	-0.17**	-0.06	-0.20**	0.16**	-0.56**	-0.43**	-0.39**	0.66**	0.51**	0.42**	0.55**

*p < 0.05;

**p < 0.01;

MAP, Mastery-Approach goals orientation; MAV, Mastery-Avoidance goals orientation; PAP, Performance-Approach goals orientation; PAV, Performance-Avoidance goals orientation; IGO, Intrinsic Goals Orientation; EGO, Extrinsic Goals Orientation; CLB, Control of Learning Beliefs.

We found significant and positive associations between mastery approach and mastery avoidance goals and all motivational components, except for test anxiety. On the contrary, performance approach and avoidance goals correlated significantly and positively with all motivational components, with the exception of intrinsic goal orientation.

### 3.3 Moderated mediation analyses

#### 3.3.1 Mastery approach goals as the predictor

For the first set of mediated moderation analyses, academic achievement was the outcome, MAP was the predictor and the motivational components were introduced as mediators. Each emotion felt during course studying was used as a moderator of the relationships between the predictor and the mediators and between the predictor and the outcome.

**FIGURE 1:**
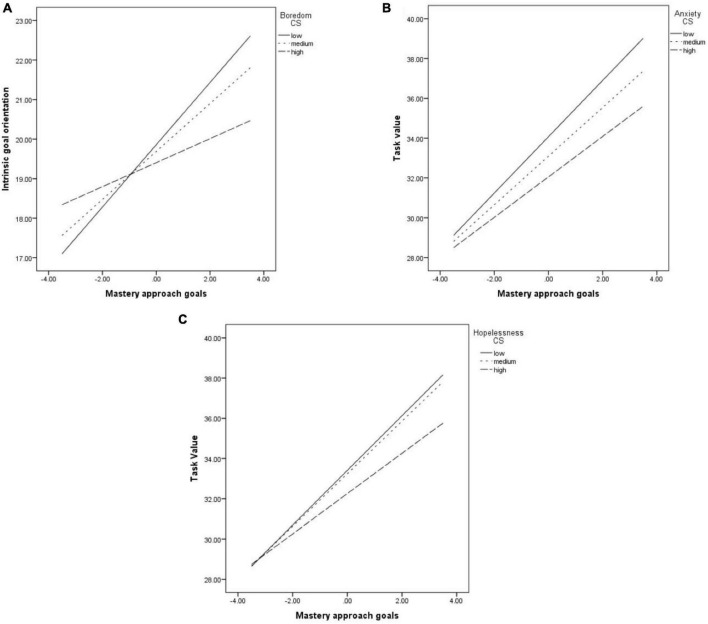
The relationship between MAV and: IGO, moderated boredom **(A)**; task value, moderated by anxiety **(B)**; task value, moderated by hopelessness **(C)**.

We found that MAP was significantly and positively related to IGO. Boredom during course studying significantly moderated this association (*b* = −0.06, *p* = 0.001). The relationship was weaker, but still significant at medium (*b* = 0.60, *p* < 0.001) and high levels of boredom (*b* = 0.30, *p* = 0.002), compared with the one at low levels of boredom (*b* = 0.78, *p* < 0.001) (see Figure 1A).

Mastery-Approach goals orientation was also significantly related to EGO, but the relationship was not moderated by any of the emotions.

The positive and significant link between MAP and task value was significantly moderated by the anxiety (*b* = −0.04, *p* = 0.01, see Figure 1B) and hopelessness (*b* = −0.05, *p* = 0.02, see Figure 1C) felt when studying. The relationship is strong at low levels of anxiety (*b* = 1.41, *p* < 0.001) and hopelessness (*b* = 1.35, *p* < 0.001, but gets weaker at medium (for anxiety, *b* = 1.22, *p* < 0.001; for hopelessness *b* = 1.30, *p* < 0.001) and low levels of the emotions (for anxiety, *b* = 1.01, *p* < 0.001; for hopelessness *b* = 1, *p* < 0.001).

**FIGURE 2:**
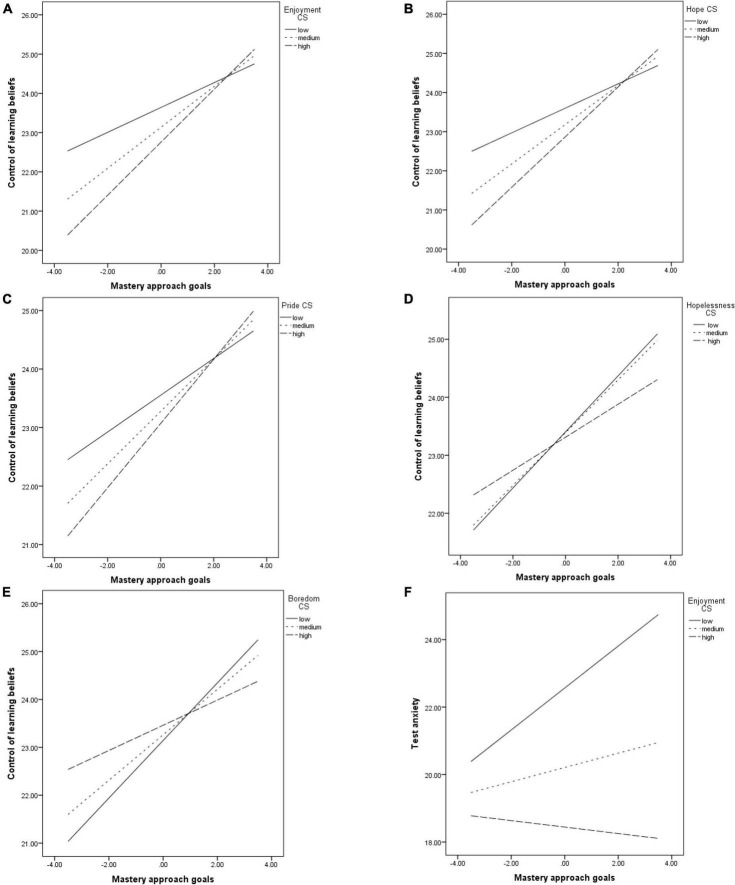
The relationship between MAP and: CLB, moderated by enjoyment **(A)**; CLB, moderated by hope **(B)**: CLB, moderated by pride **(C)**; CLB, moderated by hopelessness **(D)**: CLB, moderated by boredom **(E)**; test anxiety, moderated by enjoyment **(F)**.

The relationship between MAP and the CLB was positive and significant. However, it was moderated by the enjoyment (*b* = 0.05, *p* < 0.001, see Figure 2A), hope (*b* = 0.04, *p* = 0.006, see Figure 2B), pride (*b* = 0.03, *p* = 0.04, see Figure 2C), hopelessness (*b* = −0.02, *p* = 0.04, see Figure 2D) and boredom (*b* = −0.04, *p* < 0.001, see Figure 2E) felt while studying. Thus, when the positive emotions were involved, the link was positive, significant and stronger at medium (for enjoyment *b* = 0.52, *p* < 0.001; for hope *b* = 0.50, *p* < 0.001; for pride *b* = 0.44, *p* < 0.001) and high (for enjoyment *b* = 0.67, *p* < 0.001; for hope *b* = 0.64, *p* < 0.001; for pride *b* = 0.54, *p* < 0.001) levels of the emotions, compared to their low levels (for enjoyment *b* = 0.31, *p* < 0.001; for hope *b* = 0.31, *p* < 0.001; for pride *b* = 0.31, *p* < 0.001). When the negative emotions were involved, the link was still positive and significant, but became weaker at medium (for hopelessness *b* = 45, *p* < 0.001; for boredom *b* = 0.47, *p* < 0.001) and high levels (for hopelessness *b* = 0.28, *p* < 0.001; for boredom *b* = 0.26, *p* < 0.001) of the emotions, compared to their low levels (for hopelessness, *b* = 0.48, *p* < 0.001; for boredom *b* = 0.60, *p* < 0.001).

The relationship between MAP and self-efficacy goals was significant and positive. It was not moderated by any of the emotions felt when studying.

Enjoyment felt when studying significantly moderated the relationship between MAP and test anxiety (*b* = −0.10, *p* = 0.001). The link was significant and positive at low levels of enjoyment (*b* = 0.62, *p* < 0.001), but became non-significant at medium (*b* = 0.21, *p* = 0.14) and high levels of enjoyment (*b* = −0.09, *p* = 0.63) (see Figure 2F). Otherwise, the association was significant and positive regardless of the levels of the other variables used as moderators.

Only task value (*b* = −0.01, *p* = 0.01) and self-efficacy (*b* = 0.01, *p* = 0.001) were significantly related to academic achievement.

The direct effect of MAP on academic achievement was not significant (*b* = 0.02, *p* = 0.09). It also remained non-significant when testing it at any of the three levels of each moderator. However, the indirect effect through self-efficacy was significant and positive (*b* = 0.02, CI [01;04]). It was not moderated by any of the emotions.^1^

#### 3.3.2 Mastery avoidance goals as the predictor

For the second set of mediated moderation analyses, academic achievement was the outcome, mastery avoidance goals were the predictor and the motivational components were introduced as mediators. Each emotion felt while studying was used as a moderator of the relationships between the predictor and the mediators and between the predictor and the outcome.

**FIGURE 3:**
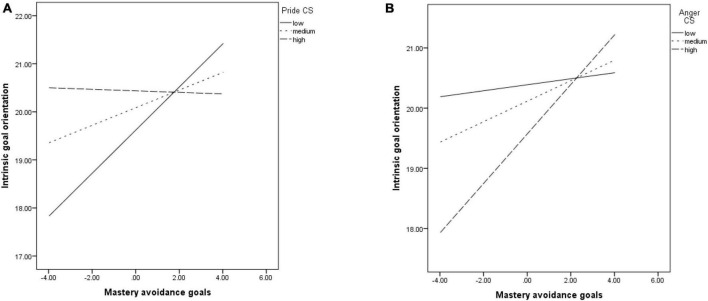
The relationship between MAV and IGO, moderated by pride **(A)**; IGO, moderated by anger **(B)**. The relationship between mastery avoidance goals and EGO was significant and positive, but it was not moderated by any of the emotions.

The positive relationship between MAV and IGO was significantly moderated by pride (*b* = −0.05, *p* = 0.006). The relationship was significant at low (*b* = 0.44, *p* < 0.001) and medium (*b* = 0.18, *p* = 0.02) levels of pride, but became non-significant at high levels of the emotion (*b* = −0.01, *p* = 0.88) (see Figure 3A). Anger also moderated the association (*b* = 0.06, *p* = 0.02), which was not significant at low levels of the emotion (*b* = 0.04, *p* = 0.64), but became significant and positive at its medium (*b* = 0.17, *p* = 0.03) and high levels (*b* = 0.41, *p* < 0.001) (see Figure 3B).

**FIGURE 4:**
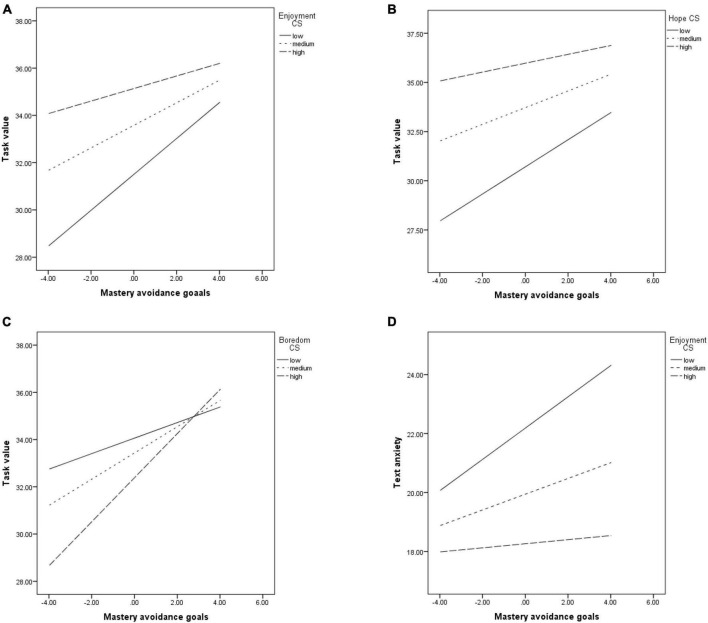
The relationship between mastery avoidance goals and: task value, moderated by enjoyment **(A)**; task value, moderated by hope **(B)**; task value, moderated by boredom **(C)**; test anxiety, moderated by enjoyment **(D)**.

Enjoyment (*b* = −0.07, *p* = 0.01, see Figure 4A), hope (*b* = −0.07, *p* = 0.02, see Figure 4B) and boredom (*b* = 0.07, *p* = 0.004, see Figure 4C) felt when studying moderated the positive link between mastery avoidance goals and task value. This association was significant at low (for enjoyment b = 0.76, *p* < 0.001; for hope *b* = 0.69, *p* < 0.001) and medium (for enjoyment *b* = 0.47, *p* < 0.001; for hope *b* = 0.42, *p* < 0.001) levels of positive emotions, but became non-significant at their high levels (for enjoyment *b* = 0.26, *p* = 0.07; for hope *b* = 0.25, *p* = 0.14). As for boredom, the relationship between mastery avoidance goals and task value was significant at low (*b* = 0.31, *p* = 0.02), medium (*b* = 0.55, *p* < 0.001) and high (*b* = 0.92, *p* < 0.001) levels of the emotion. However, it became stronger the more highly the boredom was felt.

The relationship between MAV and CLB and between MAV and self-efficacy was positive and significant throughout the models, and it was not moderated by any of the emotions.

The positive link between mastery avoidance goals and test anxiety was moderated by enjoyment (*b* = −0.06, *p* = 0.05). There was a significant relationship between the variables at low (*b* = 0.52, *p* < 0.001) and medium (*b* = 0.26, *p* = 0.03) levels of enjoyment, but it became non-significant at high levels of the emotion (*b* = 0.06, *p* = 0.69) (see Figure 4D).

The direct effect of mastery avoidance goals on academic achievement varied based on the moderator that was used. Thus, for enjoyment, the relationship was significant at low (*b* = 0.04, *p* = 0.01) and medium (*b* = 0.02, *p* = 0.02) levels of the emotion, but not at its high levels (*b* = 0.01, *p* = 0.33). Similar results were found for hope (at low levels *b* = 0.04, *p* = 0.004; at medium levels *b* = 0.02, *p* = 0.04; at high levels *b* = 0.01, *p* = 0.58) and pride (at low levels *b* = 0.03, *p* = 0.02; at medium levels *b* = 0.028, *p* = 0.01; at high levels *b* = 0.021, *p* = 0.17). On the contrary, when the negative emotions were involved, the relationship was non-significant at their low levels and became significant at their medium and high levels. Such results were found for anger (at low levels *b* = 0.01, *p* = 0.38; at medium levels *b* = 0.02, *p* = 0.03; at high levels *b* = 0.04, *p* = 0.004), anxiety (at low levels *b* = 0.02, *p* = 0.06; at medium levels *b* = 0.03, *p* = 0.01; at high levels *b* = 0.03, *p* = 0.04), for shame (at low levels *b* = 0.01, *p* = 0.18; at medium levels *b* = 0.02, *p* = 0.02; at high levels *b* = 0.05, *p* = 0.006) and for boredom (at low levels *b* = 0.01, *p* = 0.33; at medium levels *b* = 0.02, *p* = 0.02; at high levels *b* = 0.04, *p* = 0.005).

The only significant indirect effect was the one through self-efficacy. However, it was moderated by hope. It was significant at low (*b* = 0.01, CI [0.004;0.02]) and medium (*b* = 0.01, CI [0.002;0.02]) levels of hope, but became non-significant at high levels of the emotion (*b* = 0.007, CI [−0.006;0.005]). The effect remained significant when the other moderators were introduced in the models.

#### 3.3.3 Performance approach goals as the predictor

For the third set of mediated moderation analyses, academic achievement was the outcome, PAP were the predictor and the motivational components were introduced as mediators. Each emotion felt when studying was used as a moderator of the relationships between the predictor and the mediators and between the predictor and the outcome.

**FIGURE 5:**
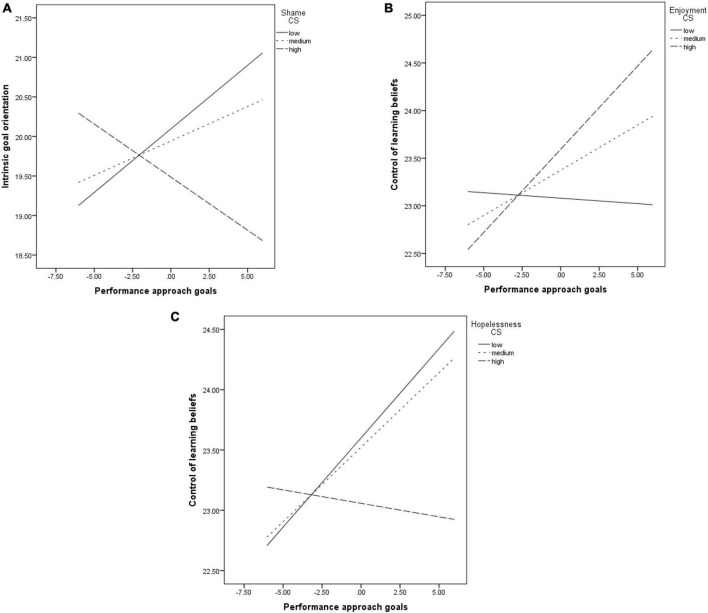
The relationship between PAP and: IGO, moderated by shame **(A)**; CLB, moderated by enjoyment **(B)**; CLB, moderated by hopelessness **(C)**.

Throughout most models, the link between PAP and IGO was not significant. It was, however, moderated by shame (*b* = −0.03, *p* = 0.01, see Figure 5A). The relationship became significant at low levels of shame (*b* = 0.16, *p* = 0.04), but not at medium (*b* = 0.08, *p* = 0.18) or high levels of the emotion (*b* = −0.13, *p* = 0.15).

PAP had significant and positive associations with EGO, task value, self-efficacy and test anxiety.

The relationship between PAP and the CLB was not significant throughout most models. It was, however, moderated by the enjoyment felt when studying (*b* = 0.02, *p* = 0.05, see Figure 5B). The link was non-significant at low levels of enjoyment (*b* = −0.01, *p* = 0.86), but became significant at medium (*b* = 0.09, *p* = 0.04) and high levels of enjoyment (*b* = 0.17, *p* = 0.01). It was also moderated by hopelessness (*b* = −0.02, *p* = 0.05, see Figure 5C). The association was significant at low (*b* = 0.14, *p* = 0.01) and medium (*b* = 0.12, *p* = 0.01) levels of hopelessness, but not at its high levels (*b* = −0.02, *p* = 0.74).

The direct effect on academic achievement was not significant (*b* = 0.02, *p* = 0.07). The significant indirect effect through self-efficacy was moderated only by pride. It became non-significant at low levels of pride (*b* = 0.006, CI [−0.006;0.01]), but remained significant at medium (*b* = 0.01, CI [0.003;0.01]) and high levels of the emotion (*b* = 0.01, CI [0.003;0.02]).

#### 3.3.4 Performance avoidance goals as the predictor

For the fourth set of mediated moderation analyses, academic achievement was the outcome, PAV were the predictor and the motivational components were introduced as mediators. Each emotion felt while studying was used as a moderator of the relationships between the predictor and the mediators and between the predictor and the outcome.

In all models, the relationships between PAV and IGO, was not significant. However, the links between PAV and EGO, self-efficacy and test anxiety were positive and significant.

**FIGURE 6:**
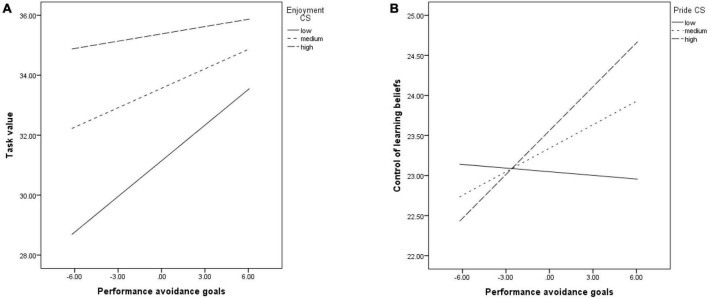
The relationship between PAV and: task value, moderated by enjoyment **(A)**; control of learning beliefs, moderated by pride **(B)**.

Enjoyment significantly moderated the positive link between PAV and task value (*b* = −0.04, *p* = 0.05). The relationship was significant at low (*b* = 0.39, *p* < 0.001) and medium (*b* = 0.21, *p* = 0.008) levels of enjoyment, but lost its significance at high levels of the emotion (*b* = 0.08, *p* = 0.48) (See Figure 6A).

Pride moderated the link between PAV and the control of learning beliefs (*b* = 0.02, *p* = 0.04). The variables were significantly related at medium (*b* = 0.09, *p* = 0.03) and higher levels (*b* = 0.18, *p* = 0.006) of pride, but not at its low levels (*b* = −0.01, *p* = 0.82). The same relationship was not significant in the other models (see Figure 6B).

The direct effect on academic achievement was not significant (*b* = 0.01, *p* = 0.36). Hope, pride and hopelessness moderated the indirect effect through self-efficacy, which was significant only at medium levels of hope (*b* = 0.006, CI [0.001;0.01]), at medium (*b* = 0.006, CI [0.001;0.01]) and high levels of pride (*b* = 0.008, CI [0.001;0.01]), and at low (*b* = 0.01, CI [0.002;0.02]) and medium levels of hopelessness (*b* = 0.01, CI [0.002;0.01]). No other emotion moderated the effect, which remained significant regardless of their levels.

## 4 Discussion

This study evaluated how achievement emotions felt while studying moderate the direct and indirect associations between specific goal orientations and academic achievement through motivational components of students. Our approach aimed to contribute to a better understanding of the underlying mechanisms and interplay of goal orientations, motivational components and emotions in academic settings that affect the performance of university students.

According to our first hypothesis, the correlational analyses indicate that both mastery-approach and performance-approach goals positively and significantly correlated with academic achievement, although the effect size was small. This result is in line with previous studies showing that students’ focus on skills, competences and knowledge development influences their academic achievement (Darnon et al., 2018; Suprayogi et al., 2019). The performance-approach goals imply overcoming others’ academic results and are associated with positive outcomes such as the use of cognitive strategies of learning (Pintrich, 2000a) and academic achievement in some studies (Goraya and Hasan, 2012; Darnon et al., 2018). However, a somewhat unexpected result is the absence of any corelation between academic achievement and intrinsic goal orientation (IGO), despite Cerasoli et al. (2014) findings that indicate in their metanalysis that exists a moderate to strong associations between intrinsic motivation and performance. As IGO represents a value component of MSLQ (Pintrich et al., 1991), it seems the subjects of our study evaluate risky, challenging and curiosity arousing learning tasks as unappropriated for obtaining good grades. The collectivistic culture (Hofstede Insights, 2020) and conformity with teachers’ expectations are both possible explanation for the irrelevance of intrinsic motivation for academic achievement, as academic results (grades).

With regard to the second hypothesis, contrary to our expectations, both mastery-avoidance and performance-avoidance goals were positively and significantly related to academic achievement. These findings contradict numerous studies that have found a negative association between both mastery-avoidance (Elliot and McGregor, 2001; Luo et al., 2013) and performance-avoidance goal orientations (Elliot and Church, 1997; Dinger and Dickhäuser, 2013; Luo et al., 2013) and academic achievement. Moreover, in our study, the positive association between performance-approach and performance-avoidance was very high (0.89), suggesting that students with performance approach goals also tend to adopt performance avoidance goals. A possible explanation of this results could be that students want to keep a good image for others and demonstrate that they can achieve good performance, avoiding to appear more incompetent than their peers. Also, mastery-approach and mastery-avoidance goals were moderately correlated, suggesting that students focus on development skills, while simultaneously avoiding misunderstanding of the content relevant for these skills. These unexpected results could be explained by Hofstede’ theory on individualistic and collectivistic cultures. As Romania has a low score on individualism (Hofstede Insights, 2020), this could explain why performance-avoidance orientation goals may be adaptative for Romanian university students. Actually, King (2016) found that cultural factors such as collectivism may explain the coexistence of both approach and avoidance learning goals orientations in those particular countries. In a previous study conducted on Chinese and Filipino students, King et al. (2014), show that individuals accommodate both achievement (mastery and performance) and social (extrinsic) goals such as affiliation, approval, concern, and status – all attributes of collectivistic cultures.

The results of the mediation analysis showed that the effect of all four goal orientation on academic achievement were mediated by academic self-efficacy (ASE). Since ASE involves one’s judgments about the capacity to project and manage desired learning goals (Bandura, 1997), it is likely that positive judgments about one’s own academic competence will enhance the goal orientation previously adopted by the individual and, in turn, their academic achievement. The mediator role played by ASE between goal orientation and academic achievement was confirmed in several similar studies (Bandalos et al., 2003; Honicke et al., 2019; Magni et al., 2021). Magni et al. (2021) found that the ASE mediated the relationships between the goal orientations and academic achievement, except for the mastery-avoidance goal orientation; however, in their longitudinal study, the role of self-efficacy as mediator was stronger for the approach orientations than for the avoidance orientations, which is in line with the original theory of Bandura (1977). Coutinho and Neuman (2008) found similar effects of ASE as mediator, except for the performance avoidance goal orientation. Honicke et al. (2019) argues that individuals with mastery-approach goals are more likely to persist in the face of adversity and to see intrinsic value in learning, compared with those with performance-approach goals. Nevertheless, in our study, the association of mastery-avoidance goals with performance was also mediated by ASE. Arguably, a possible explanation for this result is that avoiding negative learning outputs can be self-enhancing and may function as motivation for action in collectivistic cultures.

The importance of self-efficacy in learning is supported by our results. Having a higher self-efficacy also means having better self-regulation skills, which leads to learning more efficiently, with less effort, and reporting a high level of academic satisfaction (Boekaerts, 1999; Pintrich, 2000a). However, as students attempt to regulate their own learning, obstacles may arise, and thus they will have to revise their initial goals, reassess their motivation, and identify new ways to progress (Butler and Winne, 1995). In these cases, motivational regulation strategies will help students with higher self-efficacy to modify their thoughts, behaviors and emotions caused by a task, in order to stimulate the desire to complete it and overcome learning obstacles, by increasing the level of effort and engagement in academic activities and finally, their grades (Wolters, 2003; Schunk and Zimmerman, 2008; Wolters and Mueller, 2010; Trautner and Schwinger, 2020).

The moderated mediation analysis conducted using achievement emotions for testing the fourth hypothesis showed a more nuanced picture of the mediation relationships. Whereas mastery-approach indirect effect on academic achievement was not moderated by any emotion, the three mediation relationships were moderated by one or more achievement emotions.

Thus, The MAV indirect effect on academic achievement was moderated by hope. A positive and activating emotion, hope may diminish the worrying and unpleasant feelings and compensate for the avoidance dimension of MAV, and further increase beliefs in one’s own personal academic abilities. The indirect relationship of MAV with academic achievement ceased to be significant at high levels of hope, perhaps because experiencing intense, over-optimistic hope signifies that the individuals expect that positive outcomes will occur, regardless of their own actions and self-efficacy (Feldman and Kubota, 2015).

Pride is the only achievement emotion that enhances the indirect effect of PAP goal orientation on academic achievement. As a positive, retrospective, self-enhancing and output-oriented emotion, pride intensifies the association between one’s goal to outperform others and their self-worth judgments. In academic settings, pride is a positive predictor of grades and moderate the relationship between self-regulation and grades (Villavicencio and Bernardo, 2013).

PAV’s indirect effect on academic achievement was more complex. First, it was moderated by two positive emotions – hope and pride, the effect being significant only at their medium or high levels. The moderating effect of hope is consistent with the results of Feldman and Kubota (2015), where academic hope and ASE predict the students’ grade point average. A positive and activating emotion, hope can act as a buffer for the avoidance dimension from the PAV goal orientation. Hope is also an output and anticipative emotion, and can strengthen one’s self-efficacy, even when the individual is motivated by PAV goals. As for pride, being proud of past performances can help students to overcome the concerns implied by a performance avoidance goal orientation. Second, hopelessness also moderated the indirect relationship between PAV and academic achievement. Hopelessness implies negative expectations toward the future and the feeling that things are not under control. Low and medium levels of hopelessness seem to be benign for the PAV-academic achievement relationship, but when the hopelessness is too intense, the individual may withdraw from any activity, thus making the effect of PAV on achievement a non-significant one (Pekrun and Stephens, 2009).

The direct relationship between goal orientation and academic achievement was also moderated by achievement emotions. However, this was true only for mastery-avoidance goals.

First, the pattern of moderation was similar for three positive activating emotions (enjoyment, hope and pride). The relationship between the MAV goal orientation and academic achievement was stronger for the participants with low and medium levels of these emotions. In these cases, students’ have good academic performance because they want to avoid the negative consequences of not mastering the information. However, when the positive emotions felt when studying are strong, students achieve higher performance because they find enjoyment, hope and pride in learning, not because they fear the negative effects of not knowing enough.

Second, negative emotions (boredom, anxiety and anger) strengthened the relationship between the MAV goal orientation and academic achievement. Although these are emotions that are usually avoided (Rödel, 2021), they seem to reinforce learning for those with strong MAV goal orientations (Pekrun, 2018). More intense negative emotions boost the concerns already embedded in a MAV orientation, thus leading to better academic performances for students with such orientations.

One final aspect that is worth pointing out is how the achievement emotions moderated the relationships between the goal orientations and the motivational components. Relatively similar patterns were found for both mastery approach and performance approach orientations. The association of MAP with CLB was moderated by enjoyment, hope and pride. Similarly, the relationship between PAP and CLB was moderated by enjoyment. Therefore, the orientation toward complete, meaningful learning and adequate academic performance, combined with beliefs in one’s own responsibility and control over learning, are proportionally enforced by ongoing tasks increasing excitement - enjoyment, boast about past success – pride, and the expectance of positive leaning outputs – hope, respectively. These results confirm previous studies (Pekrun et al., 2006; Daniels et al., 2009), and also support Pekrun’s (2006) model, according to which students focused on competences and knowledge development are likely to feel in control of their learning and give personal value to the task; these experiences are likely to be improved by a background of positive and activating emotions.

Negative emotions such as boredom, anxiety, hopelessness and shame also moderated the relationships, which became weaker or non-significant at higher levels of the emotions. Boredom acted as a significant buffer for the link between MAP and CLB, and MAP and IGO, showing that intense boredom can overcome the students’ desire to master the information and knowledge and thus weaken the use of motivational beliefs about learning control and intrinsic motivational orientation. Anxiety moderated the association between MAP and TV, the relationship becoming weaker as anxiety increased. Both negative valence and focus of anxiety on learning outputs may explain why intense concerns and worry about learning results undermine the relationship. Hopelessness acts in similar manner: the intensity of the relationships MAP-CLB and PAP-CLB gradually decreases as hopelessness increases. This effect may be produced by the deactivating properties of hopelessness, so that individuals become gradually less confident in their control over the learning process. This negative deactivating emotion act also as a suppressor of the relationship between MAP and TV. When students experience increased hopelessness, they tend to be less engaged, considering that is difficult to maintain too ambitious MAP goals and thus become less interested in their task. Finally, shame, a negative, output emotion, associated with a sense of worthlessness and powerlessness, moderates the link between PAP and IGO. High levels of shame are likely to motivate the performance-oriented students to hide or escape the shame-inducing situation, thus decreasing the intrinsic desire to achieve the goal (Cavalera and Pepe, 2014).

The relationship between the MAP goal orientation and motivational components was moderated by three achievement emotions: enjoyment, pride and hope. The link with various motivational components (such as TV and IGO) became weaker or even non-significant at high levels of the positive emotions. Similarly, enjoyment mediated the link between PAP and TV. These results can be explained by the emotional incongruity between the anxiety of falling behind in knowledge or performance and the intense, positive emotions felt when study. Thus, high levels of positive emotions lead to good academic achievement, rather than having MAP or PAP goals. Not surprisingly, enjoyment had an inverse effect on the association between MAP and test anxiety, which became non-significant at high levels of the emotion. However, we had one rather surprising finding. Our results showed that pride, an output-oriented emotion, amplified the association of PAP goal orientation with CLB. Perhaps the concern about underperforming characteristic of PAV is slightly surpassed by remembering past successes, boosting self-confidence and strengthening the beliefs in the control of the learning process.

Negative achievement emotions also moderated the associations of the MAV goal orientation with motivational components. Interestingly, boredom gradually increased the intensity of the relationship between MAV and task value. Therefore, boredom is not always a negative emotion and could have positive benefits, such as an increase in creativity (Vodanovich, 2003). Mugon et al. (2019) point out that because boredom is unpleasant, students may be motivated to engage in an activity or material in order to reduce it. Thus, our somewhat unexpected result could be at least partially explained if we look closer to boredom as an achievement emotion. A bored student feels she is lethargic, but also restless; her mind wanders, asking herself “what if? I don’t learn as much as I can?” (an item from the MAV goal orientation scale). The student may also recall reasons to engage in the task at hand, highlighting its importance and relevance. In brief, bored students are more susceptible of reflecting on their own learning goals and to re-assess their learning priorities and values. Anger is a negative, but activating emotion and moderated the link between MAV goal orientation and IGO. This result can be explained by the fact that anger is typically associated with fight tendencies, whereas anxiety is associated with flight tendencies (Carver and Harmon-Jones, 2009; Frenzel et al., 2016). Therefore, at medium and high levels of intensity, anger replaces the worry felt by a person with MAV goals and directs him/her toward an intrinsic desire to achieve the goals.

From a theoretical standpoint, this study shows the complex role that achievement emotions play in the interplay between goal orientations, motivational components and academic achievement. However, it is still to determine the effects of negative achievement emotions (Wu and Yu, 2022). We found that self-efficacy is the only mediator that explained the relationship between goal orientation and achievement. Also, because the direct relationship between the mastery-avoidance goal orientation and academic achievement was moderated by six of the eight achievement emotions, our results suggest that this link is the most sensitive one to the influence of different achievement emotions. Moreover, this shows that the 2 × 2 goal orientation model (Elliot and McGregor, 2001) seems to be more comprehensive, at least regarding the emotional permeability of mastery-avoidance dimension in relationship to various motivational components and achievement.

Although surprisingly, both approach goals and avoidance goals had a positive relationship with academic achievement. Still, an important distinction was found, since achievement emotions moderated in different ways this association, as well as that between goal orientation and motivational components. In general, feeling positive, activating emotions when studying strengthened the relationships between the approach orientations, motivational components and academic achievement. On the contrary, feeling negative or deactivating emotions weakened the same relationships. As far as the avoidance orientations are concerned, positive and activating emotions weakened, while negative and deactivating emotions strengthened their links with motivational components and academic achievement. Some exceptions were found, such as those regarding the role of pride, which were discussed above. Alternative explanation for some inconsistent results may consists in epistemic learning-related emotions and affects as feeling of certainty or rightness, doubt, wonder or curiosity, as subject are involved in learning activities (de Sousa, 2009). These emotions, although was not directly investigated in our study, could offer valuable insights on complex relationships between avoidance orientation and motivational components.

Finally, this study shows that the cultural values can play an important part in shaping academic achievement. Unlike most previous findings, our results point toward a positive relationship between both approach and avoidance goals and achievement. Higher levels of collectivistic values might explain these results. Still, the context of these relationships is different, as shown by the moderating effects of achievement emotions.

From a practical standpoint, a global view of the moderation analyses highlights the importance of the awareness for the emotional setting of the learning process. We found more empirical support for the moderating role of achievement emotions in the relationships between mastery-goal orientations and motivational components compared to the similar role in the relationship between performance-goal orientations and motivational components. Since mastery goal orientations are more strongly associated with positive emotions (Seifert, 1995), a higher level of self-perceptions, and intrinsic motivation (Shi, 2021) and facilitate self-regulated learning (Pintrich et al., 2001), it becomes essential to create learning contexts that support positive emotions. Nevertheless, since our results show that negative emotions can strengthen the relationships between avoidance goals, motivational components and performance, practitioners should pay attention to their dynamics. While negative emotions seem to have their role in the educational process, eliciting positive emotions and directing students toward approach goals would be more appropriate. In this regard, Fritea and Fritea (2013) claim the importance of developing motivational regulation skills, since they can ameliorate or even eliminate the effects that negative emotions (e.g., boredom) have on students’ academic achievement. Finally, further exploring these relationships may suggest specific interventions in order to improve the teaching-learning process.

### 4.1 Limits

This study has several limits. The most important resides in its cross-sectional design which does not allow us to infer stronger (e.g., causal) relationships between the variables. Future studies could use a longitudinal design, thus verifying the consistency of the findings over time. Second, the convenience sample composed of social sciences university students from a single institution may be improved in future studies by randomly selecting students from various higher education institutions and faculties. Also, using a national or international sample of students would be useful and allow for inter-cultural comparison. A more expanded sample could further confirm the hypothesis that a collectivistic cultural orientation impacts the link between an avoidance goal orientation and academic achievement. Thirdly, the use of self-report instruments, despite their good psychometric proprieties, leads to other problems, such as acquiescent (tendency to strongly agree with most sentences) or reactant (e.g., disagreeing with most items of the scale) responses. Fourthly, academic achievement was measured by a single item, the self-reported, recalled value of the point average. This may be improved by considering multiple and more objective indicators of academic achievement such as class rank in class, combined with the performance in core subjects from previous years gathered from faculty records or from evaluations conducted by teachers.

## 5 Conclusion

Our results complete the existing research literature with a comprehensive analysis of the role played by each specific achievement emotion (Pekrun, 2011) as a moderator of the relationships between goal orientation, motivational components and academic achievement. In our sample of university students, we surprisingly found that both approach and avoidance goal orientations had positive relationships with academic achievement. This might be explained by the higher levels of collectivism specific to Romania. Also, self-efficacy had a significant mediation role in all the relationships. The moderation analyses showed a more complex picture. Positive and negative achievement emotions led to different patterns of associations between the other variables. This shows that higher education teachers should pay attention to the goals, emotions and learning strategies used by students, as well as to the relationship between these variables when trying to improve academic achievement.

## Data availability statement

The raw data supporting the conclusions of this article will be made available by the authors, without undue reservation.

## Ethics statement

The studies involving humans were approved by the Research Ethics Committee of the Faculty of Psychology and Education Sciences, Alexandru Ioan Cuza University, Iasi, Romania. The studies were conducted in accordance with the local legislation and institutional requirements. The participants provided their written informed consent to participate in this study.

## Author contributions

F-VF: Supervision, Conceptualization, Methodology, Writing – original draft, Writing – review and editing. RL: Conceptualization, Methodology, Writing – original draft, Writing – review and editing. OC: Conceptualization, Methodology, Formal analysis, Writing – review and editing. LC-C: Conceptualization, Writing – review and editing. RG: Conceptualization, Writing – original draft, Data curation. CO: Conceptualization, Writing – original draft, Data curation.

## Footnotes

1 The direct and indirect effects were extracted from an additional model in which we computed only the mediation analysis. A similar method was deployed for the direct effect of performance approach goals on academic achievement as well as for the direct effect of performance avoidance goals on academic achievement.

## References

[B1] Al Khatib S. A. (2010). Meta-cognitive self-regulated learning and motivational beliefs as predictors of college students’ performance. *Int. J. Res. Educ.* 27 57–71.

[B2] Alhadabi A. Karpinski A. C. (2020). Grit, self-efficacy, achievement orientation goals, and academic performance in University students. *Int. J. Adolesc. Youth* 25 519–535. 10.1080/02673843.2019.1679202

[B3] Anderman E. M. (2020). Achievement motivation theory: Balancing precision and utility. *Contemp. Educ. Psychol.* 61:101864. 10.1016/j.cedpsych.2020.101864

[B4] Bai B. Wang J. (2023). The role of growth mindset, self-efficacy and intrinsic value in self-regulated learning and English language learning achievements. *Lang. Teach. Res.* 27 207–228. 10.1177/1362168820933190

[B5] Bandalos D. L. Finney S. J. Geske J. A. (2003). A model of statistics performance based on achievement goal theory. *J. Educ. Psychol.* 95 604–616. 10.1037/0022-0663.95.3.604

[B6] Bandura A. (1977). Self-efficacy: Toward a unifying theory of behavioral change. *Psychol. Rev.* 84 191–215. 10.1037/0033-295X.84.2.191 847061

[B7] Bandura A. (1997). *Self-efficacy: The exercise of control.* New York, NY: Freeman.

[B8] Baranik L. E. Stanley L. J. Bynum B. H. Lance C. E. (2010). Examining the construct validity of mastery-avoidance achievement goals: A meta-analysis. *Hum. Perform.* 23 265–282. 10.1080/08959285.2010.488463

[B9] Bieleke M. Gogol K. Goetz T. Daniels L. Pekrun R. (2021). The AEQ-S: A short version of the achievement emotions questionnaire. *Contemp. Educ. Psychol.* 65:101940. 10.1016/j.cedpsych.2020.101940

[B10] Bipp T. van Dam K. (2014). Extending hierarchical achievement motivation models: The role of motivational needs for achievement goals and academic performance. *Pers. Individ. Differ.* 64 157–162. 10.1016/j.paid.2014.02.039

[B11] Boekaerts M. (1999). Self-regulated learning: Where we are today. *Int. J. Educ. Res.* 31 445–457. 10.1016/S0883-0355(99)00014-2

[B12] Brady M. S. (2013). *Emotional insight: The epistemic role of emotional experience.* New York, NY: Oxford University Press. 10.1093/acprof:oso/9780199685523.001.0001

[B13] Butler D. L. Winne P. H. (1995). Feedback and self-regulated learning: A theoretical synthesis. *Rev. Educ. Res.* 65 245–281. 10.3102/00346543065003245

[B14] Camacho-Morles J. Slemp G. R. Pekrun R. Loderer K. Hou H. Oades L. G. (2021). Activity achievement emotions and academic performance: A meta-analysis. *Educ. Psychol. Rev.* 33 1051–1095. 10.1007/s10648-020-09585-3

[B15] Candiotto L. (2020). Epistemic emotions and the value of truth. *Acta Anal.* 35 563–577. 10.1007/s12136-019-00416-x

[B16] Carver C. S. Harmon-Jones E. (2009). Anger is an approach-related affect: Evidence and implications. *Psychol. Bull.* 135 183. 10.1037/a0013965 19254075

[B17] Cavalera C. Pepe A. (2014). Social emotions and cognition: Shame, guilt and working memory. *Procedia Soc. Behav. Sci.* 112 457–464. 10.1016/j.sbspro.2014.01.1189

[B18] Cerasoli C. P. Ford M. T. (2014). Intrinsic motivation, performance, and the mediating role of mastery goal orientation: A test of self-determination theory. *J. Psychol.* 148 267–286. 10.1080/00223980.2013.783778 24839727

[B19] Cerasoli C. P. Nicklin J. M. Ford M. T. (2014). Intrinsic motivation and extrinsic incentives jointly predict performance: A 40-year meta-analysis. *Psychol. Bull.* 140 980–1008. 10.1037/a0035661 24491020

[B20] Çetin G. Eren A. (2022). Pre-service teachers’ achievement goal orientations, teacher identity, and sense of personal responsibility: The moderated mediating effects of emotions about teaching. *Educ. Res. Policy Pract.* 21 245–283. 10.1007/s10671-021-09303-y

[B21] Cho Y. Weinstein C. E. Wicker F. (2011). Perceived competence and autonomy as moderators of the effects of achievement goal orientations. *Educ. Psychol.* 31 393–411. 10.1080/01443410.2011.560597

[B22] Church M. A. Elliot A. J. Gable S. L. (2001). Perceptions of classroom environment, achievement goals, and achievement outcomes. *J. Educ. Psychol.* 93:43. 10.1037/0022-0663.93.1.43

[B23] Coutinho S. A. Neuman G. (2008). A model of metacognition, achievement goal orientation, learning style and self-efficacy. *Learn. Environ. Res.* 11 131–151. 10.1007/s10984-008-9042-7

[B24] Daniels L. M. Stupnisky R. H. Pekrun R. Haynes T. L. Perry R. P. Newall N. E. (2009). A longitudinal analysis of achievement goals: From affective antecedents to emotional effects and achievement outcomes. *J. Educ. Psychol.* 101:948. 10.1037/a0016096

[B25] Darnon C. Jury M. Aelenei C. (2018). Who benefits from mastery-approach and performance-approach goals in college? Students’ social class as a moderator of the link between goals and grade. *Eur. J. Psychol. Educ.* 33 713–726. 10.1007/s10212-017-0351-z

[B26] de Sousa R. (1979). The rationality of emotions. *Dialogue* 18 41–63. 10.1017/S0012217300047880

[B27] de Sousa R. (2009). Epistemic feelings. *Mind Matter* 7 139–161.

[B28] Diaconu-Gherasim L. R. Mãirean C. (2016). Perception of parenting styles and academic achievement: The mediating role of goal orientations. *Learn. Individ. Differ.* 49 378–385. 10.1016/j.lindif.2016.06.026

[B29] Dinger F. C. Dickhäuser O. (2013). Does implicit theory of intelligence cause achievement goals? Evidence from an experimental study. *Int. J. Educ. Res.* 61 38–47. 10.1016/j.ijer.2013.03.008

[B30] Dull R. B. Schleifer L. L. McMillan J. J. (2015). Achievement goal theory: The relationship of accounting students’ goal orientations with self-efficacy, anxiety, and achievement. *Account. Educ.* 24 152–174. 10.1080/09639284.2015.1036892

[B31] Dupeyrat C. Mariné C. (2005). Implicit theories of intelligence, goal orientation, cognitive engagement, and achievement: A test of Dweck’s model with returning to school adults. *Contemp. Educ. Psychol.* 30 43–59. 10.1016/j.cedpsych.2004.01.007

[B32] Dweck C. S. (1986). Motivational processes affecting learning. *Am. Psychol.* 41 1040–1048. 10.1037/0003-066X.41.10.1040

[B33] Eccles J. (1983). “Expectancies, values and academic behaviors,” in *Achievement and achievement motives: Psychological and sociological approaches*, ed. Spence J. T. (San Francisco, CA: Free man), 75–146.

[B34] Eccles J. S. Wigfield A. (2002). Motivational beliefs, values, and goals. *Annu. Rev. Psychol.* 53 109–132. 10.1146/annurev.psych.53.100901.135153 11752481

[B35] Elgin C. Z. (2008). “Emotion and understanding,” in *Epistemology and emotions*, eds Brun G. Dogluoglu U. Kuenzle D. (Hampshire: Ashgate).

[B36] Elliot A. J. (1997). “Integrating the “classic” and “contemporary” approaches to achievement motivation: A hierarchical model of approach and avoidance achievement motivation” in *Advances in motivation and achievement*, Vol. 10, eds Maehr M. L. Pintrich P. R. (Greenwich: JAI Press), 143–179.

[B37] Elliot A. J. (2005). “A conceptual history of the achievement goal construct,” in *Handbook of competence and motivation*, eds Elliot A. J. Dweck C. S. (New York, NY: Guilford Press), 52–72.

[B38] Elliot A. J. McGregor H. A. (2001). A 2× 2 achievement goal framework. *J. Pers. Soc. Psychol.* 80:501. 10.1037//0022-3514.80.3.501 11300582

[B39] Elliot A. Church M. (1997). A hierarchical model of approach and avoidance achievement motivation. *J. Pers. Soc. Psychol.* 72 218–232. 10.1037/0022-3514.72.1.218 10234849

[B40] Eum K. Rice K. G. (2011). Test anxiety, perfectionism, goal orientation, and academic performance. *Anxiety Stress Coping* 24 167–178. 10.1080/10615806.2010.488723 20503124

[B41] Feldman D. B. Kubota M. (2015). Hope, self-efficacy, optimism, and academic achievement: Distinguishing constructs and levels of specificity in predicting college grade-point average. *Learn. Individ. Differ.* 37 210–216. 10.1016/j.lindif.2014.11.022

[B42] Frenzel A. C. Pekrun R. Goetz T. Daniels L. M. Durksen T. L. Becker-Kurz B. (2016). Measuring teachers’ enjoyment, anger, and anxiety: The Teacher Emotions Scales (TES). *Contemp. Educ. Psychol.* 46 148–163. 10.1016/j.cedpsych.2016.05.003

[B43] Fritea I. Fritea R. (2013). Can motivational regulation counteract the effects of boredom on academic achievement? *Procedia Soc. Behav. Sci.* 78 135–139. 10.1016/j.sbspro.2013.04.266

[B44] Gaspard F. Wigfield A. Jiang Y. Nagengast B. Trautwein U. Marsh H. W. (2018). Dimensional comparisons: How academic track students’ achievements are related to their expectancy and value beliefs across multiple domains. *Contemp. Educ. Psychol.* 52 1–14. 10.1016/j.cedpsych.2017.10.003

[B45] Goraya F. Hasan S. S. (2012). Achievement goal orientation and academic performance in undergraduate students. *Pak. J. Soc. Clin. Psychol.* 9:27–31.

[B46] Greene B. A. Miller R. B. (1996). Infuences on achievement: Goals, perceived ability, and cognitive engagement. *Contemp. Educ. Res.* 21 181–192. 10.1006/ceps.1996.0015 8979871

[B47] Harackiewicz J. M. Barron K. E. Pintrich P. R. Elliot A. J. Thrash T. M. (2002). Revision of achievement goal theory: Necessary and illuminating. *J. Educ. Psychol.* 94 638–645. 10.1037/0022-0663.94.3.638

[B48] Hayes A. F. (2013). *Introduction to mediation, moderation, and conditional process analysis: A regression-based approach.* New York, NY: Guilford Press.

[B49] Hofer B. K. Pintrich P. R. (1997). The development of epistemological theories: Beliefs about knowledge and knowing and their relation to learning. *Rev. Educ. Res.* 67 88–140. 10.3102/00346543067001088

[B50] Hofstede Insights (2020). *Country scores.* Available online at: https://www.hofstede-insights.com/country-comparison-tool?countries=romania (accessed August 3, 2023).

[B51] Honicke T. Broadbent J. Fuller-Tyszkiewicz M. (2019). Learner self-efficacy, goal orientation, and academic achievement: Exploring mediating and moderating relationships. *High. Educ. Res. Dev.* 39 1–15. 10.1080/07294360.2019.1685941

[B52] Huang C. (2012). Discriminant and criterion-related validity of achievement goals in predicting academic achievement: A meta-analysis. *J. Educ. Psychol.* 104:48. 10.1037/a0026223

[B53] Hulleman C. S. Schrager S. M. Bodmann S. M. Harackiewicz J. M. (2010). A meta-analytic review of achievement goal measures: Different labels for the same constructs or different constructs with similar labels? *Psychol. Bull.* 136:422. 10.1037/a0018947 20438145

[B54] Kaplan A. Maehr M. L. (2007). The contributions and prospects of goal orientation theory. *Educ. Psychol. Rev.* 19, 141–184. 10.1007/s10648-006-9012-5

[B55] Keys T. D. Conley A. M. Duncan G. J. Domina T. (2012). The role of goal orientations for adolescent mathematics achievement. *Contemp. Educ. Psychol.* 37 47–54. 10.1016/j.cedpsych.2011.09.002

[B56] King R. B. (2016). Is a performance-avoidance achievement goal always maladaptive? Not necessarily for collectivists. *Pers. Individ. Differ.* 99 190–195. 10.1016/j.paid.2016.04.093

[B57] King R. B. Ganotice F. A. Watkins D. A. (2014). A cross-cultural analysis of achievement and social goals among Chinese and Filipino students. *Soc. Psychol. Educ*. 17, 439–455. 10.1007/s11218-014-9251-0

[B58] King R. B. McInerney D. M. (2014). The work avoidance goal construct: Examining its structure, antecedents, and consequences. *Contemp. Educ. Psychol.* 39 42–58. 10.1016/j.cedpsych.2013.12.002

[B59] Kitsantas A. Winsler A. Huie F. (2008). Self-regulation and ability predictors of academic success during college: A predictive validity study. *J. Adv. Acad.* 20 42–68. 10.4219/jaa-2008-867

[B60] Kosnin A. M. (2007). Self-regulated learning and academic achievement in Malaysian undergraduates. *Int. Educ. J.* 8 221–228.

[B61] Lee J. Y. Chei M. J. (2020). Latent profile analysis of Korean undergraduates’ academic emotions in e-learning environment. *Educ. Technol. Res. Dev.* 68 1521–1546. 10.1007/s11423-019-09715-x

[B62] Luo W. Aye K. M. Hogan D. Kaur B. Chan M. C. Y. (2013). Parenting behaviors and learning of Singapore students: The mediational role of achievement goals. *Motiv. Emot.* 37 274–285. 10.1007/s11031-012-9303-8

[B63] Magni F. Gong Y. Chao M. M. (2021). A longitudinal examination of the reciprocal relationship between goal orientation and performance: The mediating role of self-efficacy. *Pers. Individ. Differ.* 179:110960. 10.1016/j.paid.2021.110960

[B64] Marsh H. W. (2006). *Self-concept theory, measurement and research into practice: The role of self-concept in educational psychology. Vernon-Wall Lecture.* Leicester: British Psychological Society.

[B65] McCollum D. L. (2004). *Development of an integrated taxonomy of social goals.* State College, PA: The Pennsylvania State University.

[B66] Mega C. Ronconi L. De Beni R. (2014). What makes a good student? How emotions, self-regulated learning, and motivation contribute to academic achievement. *J. Educ. Psychol.* 106:121. 10.1037/a0033546

[B67] Mugon J. Danckert J. Eastwood J. (2019). “The costs and benefits of boredom in the classroom,” in *The Cambridge handbook of motivation and learning*, eds Renninger K. A. Hidi S. E. (Cambridge: Cambridge University Press). 10.1017/9781316823279.022

[B68] Muis K. R. Pekrun R. Sinatra G. M. Azevedo R. Trevors G. Meier E. (2015). The curious case of climate change: Testing a theoretical model of epistemic beliefs, epistemic emotions, and complex learning. *Learn. Instr.* 39 168–183. 10.1016/j.learninstruc.2015.06.003

[B69] Muwonge C. M. Schiefele U. Ssenyonga J. Kibedi H. (2019). Modeling the relationship between motivational beliefs, cognitive learning strategies, and academic performance of teacher education students. *S. Afric. J. Psychol.* 49 122–135. 10.1177/0081246318775547

[B70] Olaogun O. P. Feyijimi T. R. Hunsu N. J. (2022). “How does self-efficacy belief mediate the effects of achievement goals orientation on students’ achievement: A structural equation modeling approach,” in *Proceedings of the 2022 IEEE Frontiers in Education Conference (FIE)*, (Uppsala: IEEE), 1–4. 10.1109/FIE56618.2022.9962720

[B71] Palos R. Magurean S. Petrovici M. C. (2019). Self-regulated learning and academic performance-the mediating role of students’ achievement goals. *Rev. Cercetare Interv. Soc.* 67 234–249. 10.33788/rcis.67.15

[B72] Payne S. C. Youngcourt S. S. Beaubien J. M. (2007). A meta-analytic examination of the goal orientation nomological net. *J. Appl. Psychol.* 92:128. 10.1037/0021-9010.92.1.128 17227156

[B73] Pekrun R. (2006). The control-value theory of achievement emotions: Assumptions, corollaries, and implications for educational research and practice. *Educ. Psychol. Rev.* 18 315–341. 10.1007/s10648-006-9029-9

[B74] Pekrun R. (2011). “Emotions as drivers of learning and cognitive development,” in *New perspectives on affect and learning technologies*, eds Calvo R. D’Mello S. (New York, NY: Springer), 23–39. 10.1007/978-1-4419-9625-1_3

[B75] Pekrun R. (2018). “Emotion, lernen und leistung,” in *Bildung und emotion*, eds Huber M. Krause S. (Wiesbaden: Springer VS), 215–232. 10.1007/978-3-658-18589-3_12

[B76] Pekrun R. Stephens E. J. (2009). Goals, emotions, and emotion regulation: Perspectives of the control-value theory. *Hum. Dev.* 52 357–365. 10.1159/000242349

[B77] Pekrun R. Elliot A. J. Maier M. A. (2006). Achievement goals and discrete achievement emotions: A theoretical model and prospective test. *J. Educ. Psychol.* 98:583. 10.1037/0022-0663.98.3.583

[B78] Pekrun R. Elliot A. J. Maier M. A. (2009). Achievement goals and achievement emotions: Testing a model of their joint relations with academic performance. *J. Educ. Psychol.* 101:115. 10.1037/a0013383

[B79] Pekrun R. Goetz T. Frenzel A. C. Barchfeld P. Perry R. P. (2011). Measuring emotions in students’ learning and performance: The Achievement Emotions Questionnaire (AEQ). *Contemp. Educ. Psychol.* 36 36–48. 10.1016/j.cedpsych.2010.10.002

[B80] Pekrun R. Goetz T. Titz W. Perry R. P. (2002). Academic emotions in students’ self-regulated learning and achievement: A program of qualitative and quantitative research. *Educ. Psychol.* 37 91–105. 10.1207/S15326985EP3702_4

[B81] Pintrich P. R. (2000a). “The role of goal orientation in self-regulated learning,” in *Handbook of self-regulation*, eds Boekaerts M. Pintrich P. R. Zeidner M. (San Diego, CA: Academic Press), 451–502. 10.1016/B978-012109890-2/50043-3

[B82] Pintrich P. R. (2000b). Multiple goals, multiple pathways: The role of goal orientation in learning and achievement. *J. Educ. Psychol.* 92 544–555. 10.1037/0022-0663.92.3.544

[B83] Pintrich P. R. De Groot E. V. (1990). Motivational and self-regulated learning components of classroom academic performance. *J. Educ. Psychol.* 82:33. 10.1037/0022-0663.82.1.33

[B84] Pintrich P. R. Smith D. A. F. García T. McKeachie W. J. (1991). *A manual for the use of the motivated strategies questionnaire (MSLQ).* Ann Arbor, MI: University of Michigan, National Center for Research to Improve Postsecondary Teaching and Learning.

[B85] Pintrich P. R. Zusho A. Schiefele U. Pekrun R. (2001). “Goal orientation and self-regulated learning in the college classroom: A cross-cultural comparison,” in *Student motivation: The culture and context of learning*, eds Salili F. Chiu C.-Y. Hong Y.-Y. (Amsterdam: Kluwer Academic Publishers), 149–169. 10.1007/978-1-4615-1273-8_8

[B86] Richardson M. Abraham C. Bond R. (2012). Psychological correlates of university students’ academic performance: A systematic review. *Psychol. Bull.* 138 353–387. 10.1037/a0026838 22352812

[B87] Rödel S. S. (2021). “Negative emotions and learning,” in *Emotion–feeling–mood: Phenomenological and pedagogical perspectives*, eds Brinkmann M. Türstig J. Weber-Spanknebel M. (Wiesbaden: Springer Fachmedien Wiesbaden), 73–91. 10.1007/978-3-658-34124-4_6

[B88] Roth A. Ogrin S. Schmitz B. (2016). Assessing self-regulated learning in higher education: A systematic literature review of self-report instruments. *Educ. Assessm. Eval. Account.* 28 225–250. 10.1007/s11092-015-9229-2

[B89] Scherer K. R. (2009). The dynamic architecture of emotion: Evidence for the component process model. *Cogn. Emot.* 23 1307–1351. 10.1080/02699930902928969

[B90] Schunk D. H. Zimmerman B. J. (2008). *Motivation and self-regulated learning: Theory, research, and applications.* New York, NY: Erlbaum.

[B91] Seifert T. L. (1995). Academic goals and emotions: A test of two models. *J. Psychol.* 129 543–552. 10.1080/00223980.1995.9914926

[B92] Shi H. (2021). Examining college-level ells’ self-efficacy beliefs and goal orientation. *J. Comp. Int. High. Educ.* 13 65–82. 10.32674/jcihe.v13i2.2978

[B93] Stegers-Jager K. M. Cohen-Schotanus J. Themmen A. P. (2012). Motivation, learning strategies, participation and medical school performance. *Med. Educ.* 46 678–688. 10.1111/j.1365-2923.2012.04284.x 22691147

[B94] Suprayogi M. N. Ratriana L. Wulandari A. P. J. (2019). The interplay of academic efficacy and goal orientation toward academic achievement. *J. Phys. Conf. Ser.* 1175:012132. 10.1088/1742-6596/1175/1/012132

[B95] Tabatabaei S. S. Ahadi H. Bahrami H. Khamesan A. (2017). The effects of motivated strategies for learning questionnaire (MSLQ) on students’ cognitive and meta-cognitive skills. *NeuroQuantology* 15. 10.14704/nq.2017.15.2.1068

[B96] Tang D. Fan W. Zou Y. George R. A. Arbona C. Olvera N. E. (2021). Self-efficacy and achievement emotions as mediators between learning climate and learning persistence in college calculus: A sequential mediation analysis. *Learn. Individ. Differ.* 92:102094. 10.1016/j.lindif.2021.102094

[B97] Tang X. Lee H. R. Wan S. Gaspard H. Salmela-Aro K. (2022). Situating expectancies and subjective task values across grade levels, domains, and countries: A network approach. *AERA Open* 8, 1–16. 10.1177/23328584221117168

[B98] Thagard P. (2006). *Hot thought: Mechanisms and applications of emotional cognition.* Cambridge, MA: MIT Press. 10.7551/mitpress/3566.001.0001

[B99] Trautner M. Schwinger M. (2020). Integrating the concepts self-efficacy and motivation regulation: How do self-efficacy beliefs for motivation regulation influence self-regulatory success? *Learn. Individ. Differ.* 80:101890. 10.1016/j.lindif.2020.101890

[B100] Tze V. M. Daniels L. M. Klassen R. M. (2016). Evaluating the relationship between boredom and academic outcomes: A meta-analysis. *Educ. Psychol. Rev.* 28 119–144. 10.1007/s10648-015-9301-y

[B101] Villavicencio F. T. Bernardo A. B. I. (2013). Positive academic emotions moderate the relationship between self-regulation and academic achievement. *Br. J. Educ. Psychol.* 83 329–340. 10.1111/j.2044-8279.2012.02064.x 23692538

[B102] Vodanovich S. J. (2003). On the possible benefits of boredom: A neglected area in personality research. *Psychol. Educ. Interdiscip. J.* 40 2833.

[B103] Wigfield A. Eccles J. S. (2020). “35 years of research on students’ subjective task values and motivation: A look back and a look forward,” in *Advances in motivation science*, Vol. 7 ed. Elliot A. J. (Amsterdam: Elsevier), 161–198. 10.1016/bs.adms.2019.05.002

[B104] Wolters C. A. (1998). Self-regulated learning and college students’ regulation of motivation. *J. Educ. Psychol.* 90:224. 10.1037/0022-0663.90.2.224

[B105] Wolters C. A. (2003). Regulation of motivation: Evaluating an underemphasized aspect of self-regulated learning. *Educ. Psychol.* 38 189–205. 10.1207/S15326985EP3804_1

[B106] Wolters C. A. Mueller S. A. (2010). “Motivation regulation,” in *International encyclopedia of education*, 3rd Edn, ed. McGaw P. P. B. (Oxford: Elsevier), 631–635. 10.1016/B978-0-08-044894-7.00614-X

[B107] Wu R. Yu Z. (2022). Exploring the effects of achievement emotions on online learning outcomes: A systematic review. *Front. Psychol.* 13:977931. 10.3389/fpsyg.2022.977931 36160514 PMC9505900

[B108] Zhou Y. Wang J. (2019). Goal orientation, learning strategies, and academic performance in adult distance learning. *Soc. Behav. Pers. Int. J.* 47:e8195. 10.2224/sbp.8195

[B109] Zimmerman B. J. Schunk D. H. (2011). “Self-regulated learning and performance: An introduction and an overview,” in *Handbook of self-regulation of learning and performance*, eds Zimmerman B. J. Schunk D. H. (New York, NY: Routledge), 1–12.

